# How Medicaid and Other Public Policies Affect Use of Tobacco Cessation Therapy, United States, 2010–2014

**DOI:** 10.5888/pcd13.160234

**Published:** 2016-10-27

**Authors:** Leighton Ku, Erin Brantley, Tyler Bysshe, Erika Steinmetz, Brian K. Bruen

**Affiliations:** Author Affiliations: Erin Brantley, Erika Steinmetz, Brian K. Bruen, George Washington University, Washington, DC; Tyler Bysshe, NORC, University of Chicago, Chicago, Illinois.

## Abstract

**Introduction:**

State Medicaid programs can cover tobacco cessation therapies for millions of low-income smokers in the United States, but use of this benefit is low and varies widely by state. This article assesses the effects of changes in Medicaid benefit policies, general tobacco policies, smoking norms, and public health programs on the use of cessation therapy among Medicaid smokers.

**Methods:**

We used longitudinal panel analysis, using 2-way fixed effects models, to examine the effects of changes in state policies and characteristics on state-level use of Medicaid tobacco cessation medications from 2010 through 2014.

**Results:**

Medicaid policies that require patients to obtain counseling to get medications reduced the use of cessation medications by approximately one-quarter to one-third; states that cover all types of cessation medications increased usage by approximately one-quarter to one-third. Non-Medicaid policies did not have significant effects on use levels.

**Conclusions:**

States could increase efforts to quit by developing more comprehensive coverage and reducing barriers to coverage. Reductions in barriers could bolster smoking cessation rates, and the costs would be small compared with the costs of treating smoking-related diseases. Innovative initiatives to help smokers quit could improve health and reduce health care costs.

## Introduction

Efforts to reduce smoking by Medicaid beneficiaries are critical both to improve public health and lower health care costs in the United States, where smoking is the leading cause of preventable illness and death (1). Medicaid’s low-income beneficiaries are approximately twice as likely to smoke as the general public, and approximately 15% of Medicaid expenditures are attributable to smoking-related diseases (2). Medicaid smoking cessation initiatives can lower smoking rates, reduce cardiovascular hospital admissions, and generate a positive return on investment (3). Under the Affordable Care Act (ACA), every US state now covers at least some Medicaid tobacco cessation benefits, including medications and counseling. However, evidence indicates that only approximately 10% of Medicaid smokers receive smoking cessation medications, and use varies widely by state (4).

To understand how to bolster smoking cessation among Medicaid beneficiaries, we assessed why the use of smoking cessation therapies is higher in some states than in others. The Centers for Disease Control and Prevention (CDC), the American Lung Association (ALA), and many other organizations encourage coverage of all Food and Drug Administration (FDA)–approved cessation medications and counseling and elimination of barriers that might limit access (5–7). However, these benefits may go unused if patients and physicians are unaware that they are available or are not sufficiently engaged to attempt quitting. Fewer than half of smokers in Medicaid-managed care plans reported that their physicians offered assistance, such as medications or counseling, to quit (8).

We examined factors that could affect use of cessation medications among Medicaid beneficiaries in US states from 2010 through 2014. We examined the effects of 1) state Medicaid smoking cessation coverage policies, such as medications covered; 2) state Medicaid limitations, such as copayments, prior authorization, or requirements for counseling; 3) other state tobacco policies that may affect smoking, such as cigarette taxes or laws that restrict smoking in public places; 4) state smoking norms, based on the prevalence of smoking; and 5) other public health programs, such as the availability of medications through state-sponsored quitlines.

## Methods

We examined state-level differences in tobacco pharmacotherapy use in Medicaid, using data from 2010 to 2014. The outcome variable was the state utilization rate: the ratio of the number of prescription fills or refills paid by Medicaid for FDA-approved cessation medications divided by the estimated number of adult Medicaid smokers for each state and year, using methods previously described (4). Data for state-level drug utilization are reported under Medicaid’s drug rebate system. FDA-approved medications include nicotine replacement therapies (NRTs), including NRT gum, patches, lozenges, sprays or inhalers, and bupropion and varenicline (Chantix). These medications ease withdrawal from cigarettes and are effective, albeit imperfect, in boosting success in quitting. Many NRT products are sold over the counter, and Medicaid will pay for over-the-counter medications when prescribed by a clinician. Bupropion may be prescribed for smoking cessation, depression, or both. Because claims data do not indicate the reason it was prescribed, we created 2 versions of the variable: one version that includes all FDA-approved medications including bupropion (150 mg, twice per day, which is the recommended dosage), and one that excludes it.

There were 255 state-year observations for the 50 states and the District of Columbia over 5 years. Our analyses considered the possible effects of several factors that may affect the use of cessation medications by Medicaid smokers including Medicaid benefit policies, other state tobacco policies, smoking norms, and public health programs.

### Medicaid benefit policies

These data were based on surveys and interviews conducted by the ALA for CDC (9); Anne DiGilulio of ALA shared unpublished 2013 data. The first variable was whether the state Medicaid agency covered all 3 categories of medications: NRTs, bupropion, and varenicline. (If the state covered any NRT product, it counted for this category. Almost all states cover NRT gum and patches, but they vary in coverage of less widely used sprays, inhalers, and lozenges.) We created 2 binary versions, one version indicating that the policy applies to all Medicaid populations, including all managed care enrollees, and an alternative version indicating that either all populations were covered or that Medicaid managed care organizations (MCOs) had flexibility to determine specific policies. Both versions equaled 0 if there was no coverage or coverage only for pregnant women. Most nonelderly, nondisabled Medicaid adults are enrolled in MCOs. States may require that MCOs follow uniform state-based policies but often leave some flexibility in benefit design to MCOs. Although policies for pregnant women are important and the ACA requires that comprehensive cessation services be available for them, our data included pregnant women as part of total utilization. Data about policies only for pregnant women, who are a small fraction of beneficiaries, were not included in this analysis.

Other Medicaid policies examine restrictions on use of cessation medications: whether the state charges nominal copayments, requires prior authorization before prescription, limits the duration of coverage (eg, number of months of coverage), or permits medications only if counseling is also received. Medicaid cannot require cost-sharing for most children and pregnant women (10) and, under the ACA, should cover tobacco cessation for adults enrolled under Medicaid expansions without cost-sharing. For each policy, we developed binary variables that indicated whether the state had the restriction for all populations (except those with mandatory exclusions, such as cost-sharing exclusions). A variant of the binary variable also included states in which policies could vary for MCOs.

### Other state tobacco policies, smoking norms, and public health programs

We included the value of cigarette taxes per pack for each state in the previous year (11). Taxes increase cigarette prices and discourage smoking, especially among youth (1). The second variable was state smoke-free laws that restrict smoking in public areas, specifically restaurants, bars, and nonhospitality workplaces (12) (coded as 0 for no restrictions, 1 for restrictions in 1 or 2 areas, and 2 for restrictions for all 3 areas). Smoke-free laws are designed to reduce secondhand smoke exposure, and they also signal public unacceptability of smoking.

Regional variations exist in smoking prevalence, suggesting differences in social norms about smoking. We used 2010 through 2014 data from the Behavioral Risk Factor Surveillance System to assess the percentage of a state’s adult population that currently smokes each year (13).

All health agencies have quitlines, toll-free services that offer telephone counseling and sometimes free or discounted medications (eg, a voucher that can be redeemed for NRT medications). We included whether quitlines offer free or discounted medications (NRTs, bupropion, or varenicline) (14). Most quitlines offer NRT gum or patches; few offer bupropion or varenicline. The National Cancer Institute offers a national quitline, and some insurers may also offer quitlines. However, our models included data for the state quitlines only. (The federal quitline does not offer medications, but it may refer a caller to a state quitline.)

### Analytic strategy

We used longitudinal panel estimation methods to conduct fixed-effects regression models of how changes in policies affect use of cessation medications, controlling for both state-level and year-related effects. The general model is equivalent to:

U_st_ = β_0_ + βX_st_ + βSt + βT + ε_st_

where U_st_ is the utilization rate (prescriptions per Medicaid smoker) in state S and year T, X_st_ is a vector of independent variables in each state and year, St is a time-invariant dummy for each state, T is the year, βs are the estimated coefficients and ε_st_ is the residual. Fixed-effects models, compared with random-effects models, are appropriate because the data include all states, not a sample. We used robust standard errors with Huber-White estimators to adjust for heteroscedasticity.

We tested the effects of policies, both single and multiple policies, on medication utilization rates, using 2-way fixed effects to control for state and year. Controlling for each state nets out the effects of unmeasured state characteristics — such as racial, educational, or age composition or region or status as a tobacco-producing state — which change very little over the years but that may also be associated with smoking or cessation behaviors on a cross-sectional basis. Controlling for year effects limits the effects of national trends, such as changes in federal implementation of the ACA or the Family Smoking Prevention and Tobacco Control Act or changes in the nontax prices of cigarettes.

Our analyses treated all states as having equal weight. We considered weighting by the number of Medicaid smokers in each state but decided against it, because it would let results from 2 larger states (New York and California) have weight equal to 28 smaller states. Significance was set at *P* < .10.

## Results

Table 1 summarizes trends in relevant factors, presenting data for the first and last years, 2010 and 2014. The mean use of Medicaid tobacco cessation medication changed little nationally, although state levels varied widely. From 2010 to 2014, there were modest increases in the percentage of states that covered all categories of cessation medications, with smoke-free laws, and whose quitlines offered free or discounted medications. Mean cigarette taxes rose, restrictions on Medicaid cessation coverage policies became less common, and fewer states required copayments or counseling. In models testing individual policy variables, only 2 variables were significant; requiring counseling to get medications had a negative effect on use (*P* = .02), and whether the state covered all types of medications increased use (*P* = .04) (data not shown). No significant results were found for the other variables.

**Table 1:** Summary Data for Key Variables Among States, Use of Tobacco Cessation Medications Among Medicaid Smokers, United States, 2010 and 2014

Variable	Value (n = 51)	
	2010	2014
**Medicaid smoking cessation utilization rate, mean (SD)**		
Annual fills or refills (all categories), per Medicaid smoker	0.192 (0.133)	0.189 (0.096)
Annual fills or refills (excluding bupropion), per Medicaid smoker	0.089 (0.090)	0.087 (0.063)
**Adults who are current smokers, mean % (SD)**	17.9 (3.4)	18.5 (3.5)
**Prior year cigarette taxes per pack, mean $ (SD)**	1.29 (0.80)	1.49 (0.95)
**Percentage of states in which Medicaid . . .**		
Covers all categories of cessation medications, all populations	62.8	74.5
Covers all categories of cessation medications or permits variation across managed care organizations	86.3	89.0
Requires copayments	54.9	47.1
Requires prior authorization	41.2	41.2
Limits duration of benefits	47.1	47.1
Requires counseling to receive cessation medications	31.4	23.5
**State smoke-free laws, % of areas restricted**		
None	29.4	27.5
1 or 2	27.5	23.5
3	43.1	49.0
**State quitline offers free or discounted medications, %**	76.5	90.2

We used 4 multivariate models to examine the effects of changes in state policies and characteristics on state-level use of Medicaid tobacco cessation medications. Model 1 examines Medicaid policies that apply uniformly for all populations. Model 2 loosens criteria to include states in which MCOs can vary policies, as well as states with uniform policies. Even when MCOs have flexibility, their policies are often the same as state fee-for-service policies (15). We used 2 additional models (Models 3 and 4) to examine Medicaid utilization rates for tobacco cessation medications excluding bupropion and including only NRTs and varenicline. Because it is uncertain if bupropion was prescribed for smoking cessation or not, its inclusion might obscure the effect of smoking-related policies. These models are more conservative because they only include medications for which smoking cessation is the primary indication. Model 3 focuses on policies consistent for all populations, while Model 4 also includes policies that may vary across MCOs.

Table 2 presents the results of Models 1 and 2 in which the dependent variable is the state utilization rate for all FDA-approved medications in a year. In both models, requiring counseling to get cessation medications reduced utilization rates (coefficient = −0.044 for Model 1; coefficient = −0.057 for Model 2; both *P* < .01). Given that the mean cessation utilization rate was 0.189 fills/refills per smoker in 2014, these policies have an average effect of reducing utilization by 23% to 30%, controlling for all other factors. Covering all cessation medications was marginally associated with higher utilization (*P* = .051 in Model 1, *P* = .102 in Model 2), suggesting increases of approximately 24% to 34%. Despite the uncertainty of how completely the MCOs covered or did not cover benefits, the estimated coefficients are generally much larger in Model 2 than Model 1, suggesting that Model 2 captures effects for policies that affect the large share of beneficiaries in managed care, while Model 1 failed to capture some of those effects.

**Table 2:** Two-Way Fixed Effects Regression Models of Factors Affecting Utilization Rates of All Types of Tobacco Cessation Medications Among Medicaid Smokers (n = 255), United States, 2010–2014

State Variable	Model 1: Medicaid Policies, All Populations	Model 2: Medicaid Policies, All Populations, Varies by Managed Care Plan
	Coefficient (Standard Error)	
**Medicaid policies**		
Covers all types of medications	0.045 (0.022)^a^	0.065 (0.039)
Requires copayments	0.013 (0.027)	0.002 (0.034)
Requires prior authorization	0.014 (0.016)	0.023 (0.018)
Limits duration of benefits	−0.001 (0.011)	0.007 (0.018)
Requires counseling	−0.044 (0.016)^b^	−0.057 (0.017)^b^
**Other tobacco policies**		
Prior year cigarette taxes	0.009 (0.019)	0.003 (0.017)
No. of smoke-free restrictions	0.015 (0.023)	0.031 (0.032)
**Social norms: % of adults currently smoking**	−0.006 (0.005)	−0.006 (0.005)
**Public health programs: quitline offers medications**	−0.012 (0.020)	−0.004 (0.020)
***R* ^2^**		
Within	0.165	0.159
Between	0.353	0.324
Total	0.323	0.298

a *P* < .10.

b *P* < .01

Table 3 presents the results of Models 3 and 4, which examined Medicaid utilization rates for tobacco cessation medications, excluding bupropion and including only NRTs and varenicline. The results were similar to those of Models 1 and 2: requiring counseling was significantly associated with a reduction in cessation utilization, and coverage of all types of cessation medications was associated with higher utilization. The results of Model 4 may be the best specified of all 4 models; the coefficients were larger than those in Model 3, indicating that it captured effects from managed care populations. It has the highest within-*R* ^2^ of all 4 models, indicating the best fit of the effect of policy changes, controlling for state- and time-invariant measures.

**Table 3:** Two-Way Fixed Effects Regression Models of Factors Affecting Utilization Rates of Nicotine Replacement Therapies and Varenicline, Excluding Bupropion, Among Medicaid Smokers, United States, 2010–2014

State Variables	Model 3: Medicaid Policies, All Populations	Model 4: Medicaid Policies, All Populations, Varies by Managed Care Plan
	Coefficient (Standard Error)	
**Medicaid policies**		
Covers all types of medications	0.038 (0.019)^a^	0.070 (0.032)^b^
Requires copayments	0.020 (0.023)	0.013 (0.028)
Requires prior authorization	0.017 (0.013)	0.015 (0.013)
Limits duration of benefits	−0.001 (0.009)	0.003 (0.014)
Requires counseling	−0.043 (0.014)^c^	−0.045 (0.014)^c^
**Other tobacco policies**		
Prior year cigarette taxes	0.005 (0.015)	0.002 (0.014)
No. of smoke-free restrictions	0.009 (0.015)	0.025 (0.024)
**Social norms: % of adults currently smoking**	−0.002 (0.003)	−0.003 (0.003)
**Public health programs: quitline offers medications**	−0.011 (0.013)	−0.003 (0.014)
***R* ^2^**		
Within	0.198	0.204
Between	0.369	0.340
Total	0.333	0.311

a *P* < .10.

b *P* < .05.

c *P* < .01

## Discussion

This analysis is the first to identify that requiring counseling to get cessation medications sharply lowers their use. Previous research found that quitting can be more successful when patients use both counseling and medications, although either alone are also effective (16,17). Greene et al found that states with Medicaid policies permitting both counseling and pharmacotherapy had more quit attempts and successful quit attempts (18). Medicaid agencies may have sought to be helpful by requiring counseling to ensure that patients get a stronger combination of interventions. However, the policy appears to have unintended consequences of reducing medication use by about one-quarter to one-third. Moreover, counseling may be uncommon in Medicaid. For example, in Arkansas only 23% of Medicaid patients who received tobacco cessation benefits received counseling (19). State Medicaid agencies and MCOs should consider recommending that patients seek both medications and counseling, but not requiring one to get the other.

Broader Medicaid coverage of FDA-approved medications appeared to be weakly associated with greater use of medications. Guidelines, including the federal *Healthy People 2020* objectives, have recommended comprehensive Medicaid coverage of evidence-based therapy in all states (20). These findings show that broader coverage stimulates greater cessation efforts. Offering more choices can enhance the demand for medications and increases the likelihood of finding medications meeting patients’ needs or clinician’s judgment. NRTs and bupropion are relatively inexpensive medications. Though varenicline is more expensive, it is not a high-cost drug, particularly because it is usually prescribed for brief periods, and research indicates that it is more effective for quitting (21). The total amount Medicaid spends on tobacco cessation medications is well below 1% of the cost to Medicaid of smoking-related diseases (4); Medicaid programs should not be attempting to save money in this area.

In this analysis, nominal copayments were not related to utilization, in contrast to findings of earlier research. One study found that Medicaid copayments were associated with fewer successful quit attempts (11), and another found that Medicaid copayments diminished the extent to which postpartum women fill their prescriptions (22). A broad literature indicates that copayments lower use of preventive health services, such as the use of drugs for hypertension or diabetes (23). Perhaps copayments have little effect in reducing use of tobacco cessation medications because those seeking help are motivated to quit and the duration of use is usually brief (2 or 3 months); therefore, the out-of-pocket costs may be small relative to the expected benefit.

Nonetheless, many organizations recommend against Medicaid policies such as copayments, prior authorization, and limits of duration of benefits that could restrict access to cessation medications (5–7). Although the analyses did not result in significant effects of these policies, common sense suggests that they could limit access for some, and there is no compelling reason for states to adopt or retain restrictive policies, given the low cost of tobacco cessation therapy.

The lack of effect of cigarette taxes or smoke-free laws was surprising. Previous research has consistently found that these are 2 of the most effective strategies to reduce smoking (1). One possible explanation could be that taxes and smoke-free policies could primarily affect initiation or prevalence of smoking, while our study is measuring quit attempts among those who are already smoking. An earlier study also failed to find a significant relationship between state cigarette taxes and Medicaid quit attempts (24). Nonetheless, it seems logical that higher taxes would persuade more low-income Medicaid beneficiaries to try to quit to ease financial burdens. Many people who may be affected by proposed federal regulations to restrict smoking in public housing (25) will be Medicaid beneficiaries, and efforts to facilitate cessation among public housing clients will be needed.

The study has several limitations. We examined 2 potential sources of error — measurement of Medicaid policies (all populations vs all populations plus managed care variation) and of tobacco cessation medications (with and without bupropion) — using alternative specifications and found consistent results. We were concerned that medications paid by Medicaid may exclude some Medicaid beneficiaries who get medications through programs such as quitlines. However, because quitline variables were not significant, our measures do not appear to be biased. There may be additional measurement error because of potential problems in state reports of drug utilization or because of our methods of estimating the number of Medicaid smokers in each year. Finally, the unit of analysis was the state rather than the individual or community. We plan to conduct individual-level models to examine outcomes, such as quit attempts, but could only exploit the analytic benefits of panel models in controlling for unmeasured state- or year-related characteristics with state-level data.

Although these analyses examined the effects of certain well-defined policies and factors, a shortcoming was that we could not examine other hard-to-measure policies such as state, local, or managed care initiatives to bolster Medicaid tobacco cessation, whether through communications to the public or to clinicians, or federal campaigns offered across the country. For example, Massachusetts initiated a campaign, developed by the state health and Medicaid agencies, which led to lower smoking prevalence, reduced hospitalizations, and Medicaid savings (3,26). Other innovative state initiatives have been documented (27). However, the diverse nature of these initiatives made counting and classification difficult. A multiyear study in Arkansas found that Medicaid coverage of tobacco cessation increased utilization, but the effects waned over time; the authors concluded that other types of efforts are needed to continue to motivate smokers and to limit barriers to use of cessation therapies (19).

A methodological strength of this study is that longitudinal panel analysis offers stronger evidence of the causal impact of Medicaid policies on cessation utilization than do simpler cross-sectional studies. Two-way fixed effects models measure how changes in state policies or characteristics lead to changes in medication use, netting out effects of unmeasured state characteristics and secular trends. Also, our approach examined an array of policy and social factors, within and outside of Medicaid, that might affect the extent to which Medicaid beneficiaries try to quit smoking.

The ultimate challenge is to empower patients and providers to increase awareness of cessation benefits and to enhance motivation and supports to undertake the difficult challenge of quitting, to improve health and to reduce health care costs. Quit attempts often end in relapses to smoking, and innovative strategies are needed to attain long-term success. Medicaid covers tens of millions of high-risk, low-income Americans, and Medicaid expansions are increasing coverage of low-income smokers. The cost of smoking cessation treatments is minute compared with the cost of smoking-related diseases in Medicaid. Stronger efforts can both improve health and save money. An important first step is for states to broaden Medicaid coverage of all approved medications and eliminate barriers impeding access and utilization. The long-term challenge will be for Medicaid to collaborate with public health agencies, managed care plans, health systems, and clinicians to develop and implement more effective strategies to help smokers to quit and to monitor the effectiveness of these efforts.
